# *CTNNB1* mutation represents a recurrent driver molecular alteration in a subset of endometrial stromal tumors

**DOI:** 10.1007/s00428-026-04420-6

**Published:** 2026-01-28

**Authors:** Pavel Dundr, Eliška Radová, Jan Hojný, Nikola Kudrnová, Michaela Kendall Bártů, Kristýna Němejcová, Marta Kalousová, Filip Frühauf, David Cibula, Radoslav Matěj, W Glenn McCluggage, Ivana Stružinská

**Affiliations:** 1 Department of Pathology, First Faculty of Medicine, Charles University and General University Hospital in Prague, Studničkova 2, 12800 Prague 2, Czech Republic; 2 Institute of Medical Biochemistry and Laboratory Diagnostics, First Faculty of Medicine, Charles University and General University Hospital in Prague, Prague, Czech Republic; 3 Gynecologic Oncology Centre, Department of Gynaecology, Obstetrics and Neonatology, First Faculty of Medicine, Charles University and General University Hospital in Prague, Prague, Czech Republic; 4 Department of Pathology, Charles University, 3rd Faculty of Medicine, University Hospital Kralovske Vinohrady, 10034 Prague, Czech Republic; 5 Department of Pathology and Molecular Medicine, Third Faculty of Medicine, Charles University, Thomayer University Hospital, Prague, Czech Republic; 6 Department of Pathology, Belfast Health and Social Care Trust, Belfast, UK

**Keywords:** Uterine tumor, Low-grade endometrial stromal sarcoma, *CTNNB1* gene mutation, Endometrial stromal tumor with whorling

## Abstract

Low-grade endometrial stromal sarcomas (LGESS) and endometrial stromal nodules represent a distinct entity with characteristic morphology, immunohistochemical profile, and molecular features. It has been suggested that the activation of the Wnt signaling pathway is a potential driver in some of these tumors. Approximately 70–75% of LGESS are characterized by non-random recurrent fusions mostly involving *JAZF1* and *PHF1* genes. Although the exact mechanism remains unclear, activation of the Wnt signaling pathway appears to be related to the functional deregulation of chromatin remodeling complexes caused by these fusion proteins. However, knowledge about other possible mechanisms and recurrent molecular alterations occurring at the DNA level in LGESS remains limited. We report three cases of LGESS in patients aged 39, 49, and 50, lacking recurrent fusions but harboring *CTNNB1* mutations in exon 3 as the sole detectable molecular alteration. One case had morphological features of typical LGESS (in some areas with perivascular whorling, which was not a dominant feature), the second case showed features of the fibroblastic variant of LGESS, and the third case comprised a LGESS with a peculiar morphology with diffuse whorling morphologically identical to the recently described entity endometrial stromal tumor with *GREB1::CTNNB1* fusion. RNA-Seq-based clustering analysis of the three cases and a set of 193 uterine tumors showed that the *CTNNB1*-mutated cases clustered near LGESS cases. This study adds to the growing body of evidence that *CTNNB1* mutations represent a driver molecular event in a small subset of endometrial stromal tumors. Moreover, the results of our study suggest that tumors with *CTNNB1* mutation and *GREB1::CTNNB1* fusion may exhibit identical morphology and potentially represent the same category of tumor. Characterization and reporting of additional cases are needed to determine whether these are part of the spectrum of low-grade endometrial stromal tumors (either endometrial stromal nodule or LGESS) or represent a separate category of tumors characterized by *CTNNB1* alteration as a driver event.

**Supplementary Information:**

The online version contains supplementary material available at 10.1007/s00428-026-04420-6.

## Introduction

Low-grade endometrial stromal sarcomas (LGESS) and endometrial stromal nodules (ESN), most commonly occurring in the uterine corpus, represent a distinct entity with characteristic morphological, immunohistochemical, and molecular features. It has been shown that non-random recurrent fusions are present in 70–75% of these tumors, and most of these alterations appear to represent driver events [1–4]. Most fusions occurring in LGESS involve genes associated with chromatin remodeling complexes that regulate transcription, namely *JAZF1* (with *SUZ12*,* PHF1*,* CDKN1A*,* SYNGAP1*) and *PHF1* (with *JAZF1*,* BRD8*,* EPC1*,* EPC2*,* ING3*,* MEAF6*,* MBTD1*) [3, 5, 6]. Fusions involving these genes account for approximately 85–90% of all gene fusions identified in LGESS [3]. Knowledge regarding gene mutations in LGESS remains limited because most studies have focused only on gene rearrangements. However, current evidence suggests that activation of the Wnt signaling pathway is a potential driver event in some LGESS [7, 8]. This hypothesis is supported by the observation that a substantial proportion of LGESS (approximately 60%) show aberrant nuclear expression of β-catenin, a component of the membranous adhesion system that also plays a crucial role as a central activator in the canonical (β-catenin-dependent) Wnt pathway [9, 10]. It has been proposed that fusions typical for LGESS can influence the Wnt pathway, resulting in abnormal Wnt signaling [7, 8]. Although the exact mechanism remains unclear, it appears to be related to functional deregulation of chromatin remodeling complexes by fusion proteins [8]. For example, the *JAZF1::SUZ12* fusion protein has been shown to disrupt the polycomb repression complex 2 (PCR2) and impair its transcriptional repression activity [7]. A subsequent study confirmed this finding and demonstrated overexpression of other genes regulated by *SUZ12* [8]. The authors suggested that based on the overexpression of a significant number of Wnt-related genes regulated by *SUZ12*, gene fusions characteristic for LGESS may play an initiating role in activating the Wnt signaling pathway. However, this activation appears to be largely unrelated to primary alterations in the *CTNNB1* gene, as no increased mRNA expression of *CTNNB1* was observed in their study. To date, only one case of LGESS with a *CTNNB1* mutation has been reported in the literature [11].

In this study, we report three LGESS lacking recurrent fusions, but harboring *CTNNB1* mutations; this was the only detectable molecular alteration in two of the cases, the third case harboring additional alterations. Two of our cases showed features of LGESS (a usual variant with minor areas of whorling, and a fibroblastic variant), while the third case showed morphology characterized by diffuse whorling, which was recently described in endometrial stromal tumors with *GREB1::CTNNB1* fusion [12–14]. This study adds to the evidence that *CTNNB1* mutations are the driver molecular event in a small subset of endometrial stromal tumors (EST). Moreover, the results of our study suggest that low-grade ESTs with *CTNNB1* mutation and with whorling and *GREB1::CTNNB1* fusion potentially represent the same category of tumor, which could belong to the spectrum of EST, either ESN or LGESS, or represent a separate category of neoplasms characterized by *CTNNB1* alterations (mutation or fusion) as a driver event. In reporting these cases, we review the literature on *CTNNB1* mutations in EST and also tumors with whorling and *GREB1::CTNNB1* fusion.

## Material and methods

The study involved three cases of uterine EST with *CTNNB1* mutation. One patient was drawn from a cohort of 128 LGESS cases that were analyzed as a part of the Rare Gynecologic Sarcoma (REGYS) project [2]. The two other patients were identified in the archive files of our institutions and were picked up during routine diagnostic next generation sequencing (NGS) testing.

For comparison of RNA-Seq expression patterns, data from previous studies, including 87 fusion-positive LGESS, 20 fusion-positive and 20 fusion-negative high-grade endometrial stromal sarcomas (HGESS), 45 undifferentiated uterine sarcomas (UUS), 20 fusion-positive uterine tumors resembling ovarian sex cord tumors (UTROSCT), and one case of *GREB1::CTNNB1* fusion-positive tumor were used [13].

### Immunohistochemical analysis

Immunohistochemistry was performed using 4-µm-thick sections of formalin-fixed and paraffin-embedded (FFPE) tissue following standard protocols. The antibodies used were estrogen receptor (clone SP1, 1:200, Zytomed Systems GmbH, Berlin, Germany), progesterone receptor (clone 16, 1:100, Novocastra, Leica Biosystems, Wetzlar, Germany), smooth muscle actin (clone 1A4, 1:1600, Dako, Glostrup, Denmark), desmin (clone D33, 1:200, Dako), caldesmon (clone H-cald, 1:800, Santa Cruz Biotechnology, Dallas, TX, USA), calponin (clone CALP, 1:400, Dako), CD10 (clone 56C6, 1:50, Novocastra), IFITM1 (polyclonal rabbit, 1:400, Abcam, Cambridge, UK), transgelin (clone 2A10C2, 1:400, BioSB, Santa Barbara, CA, USA), WT-1 (clone 6F-H2, 1:200, BioSB), β-catenin (clone 14, 1:100, BioSB), and β-catenin (clone 1, 1:400, Dako).

### NGS analyses

The genomic DNA and total RNA were simultaneously isolated from FFPE tissue and used for DNA and RNA NGS analyses.

#### DNA

The targeted-capture NGS of DNA (covering 783 genes or gene parts; 2411 kbp of target sequence; 1978 kbp of coding sequence) was performed. The DNA library preparation was carried out as described previously [12]. Libraries were paired-end sequenced on NextSeq 2000 instruments (Illumina). Bioinformatic evaluation and analysis of raw fastq files were performed using the constructed bioinformatic pipeline in CLC Genomics Workbench v23.0.5 (GW) and the GRCh38 genome build as described previously in detail [2].

#### RNA

A ribodepletion-based whole-transcriptome RNA-Seq (transcriptome RNA-Seq) approach for the detection of all potential fusion events and mRNA expression was performed.

The constructed transcriptome libraries with unique molecular index (UMI) adapters were paired-end sequenced on the NextSeq 2000 instrument (Illumina) using the P2200/300 cycles reagent kit. Data were processed using a custom RNA-Seq bioinformatic pipeline, as described in detail previously [2, 15].

RNA-Seq-based clustering was analyzed using Qlucore Omics Explorer 3.10 (Qlucore AB) as follows: total exon reads of all samples were normalized using variance stabilizing transformation (VST) approach; normalized data were statistically evaluated using DESeq2 module and Multi Group Likelihood Ratio Test (LRT) where groups were characterized as *CTNNB1*_mutated (3 samples); *CTNNB1*_fused (1 sample); fusion-driven LGESS (87 samples); *BCOR*-driven HGESS (10 samples), *YWHAE*-driven HGESS (10 samples); HGESS without driver fusion (20 samples); UUS (45 samples); and fusion-driven UTROSCT (20 samples). Optimal cluster separation was achieved by UMAP plotting of 1670 most differently expressed genes (*p* < 0.0001).

The RNA-Seq expression patterns of *CTNNB1*-altered cases were compared with 87 LGESS using the “Differential Expression in Two Groups” module in CLC Genomics Workbench v25 (CLC; Qiagen) software. Only statistically significant gene expressions with TPM mean in max group > 5 and fold change > 2 were further evaluated and Volcano plotted as described previously [15]. Individual TMM-normalized expression data (counts per million; CPM) were plotted using violin plots and statistically evaluated by Mann–Whitney test in GraphPad Prism 9.5.1 (GraphPad Software, LLC).

## Results

### Clinical features

#### Case 1

A 50-year-old woman underwent hysterectomy with bilateral salpingo-oophorectomy due to a suspicious finding in a curettage specimen performed for abnormal uterine bleeding at another institution. The patient did not receive any systemic adjuvant treatment. Currently, 12 years after diagnosis, she shows no evidence of disease.

#### Case 2

A 39-year-old woman underwent partial resection of a uterine fibroid in another country. The biopsy result was not available, but according to the patient, the lesion was benign. She presented to our institution with recurrent uterine bleeding. Ultrasonography revealed a tumor measuring 55 mm in its greatest dimension. She underwent laparoscopic myomectomy, which, after a diagnosis of LGESS, was followed by hysterectomy with bilateral salpingectomy 6 weeks later. The patient did not receive any systemic adjuvant treatment. Currently, 10 months after diagnosis, she shows no evidence of disease.

#### Case 3

A 49-year-old woman underwent hysterectomy with bilateral salpingectomy due to clinical diagnosis of fibroids recently complicated by massive uterine bleeding. The postoperative course was uneventful, but follow-up for this patient is not yet available as the patient is currently only one month after surgery.

### Pathologic features

#### Case 1

**Fig. 1:**
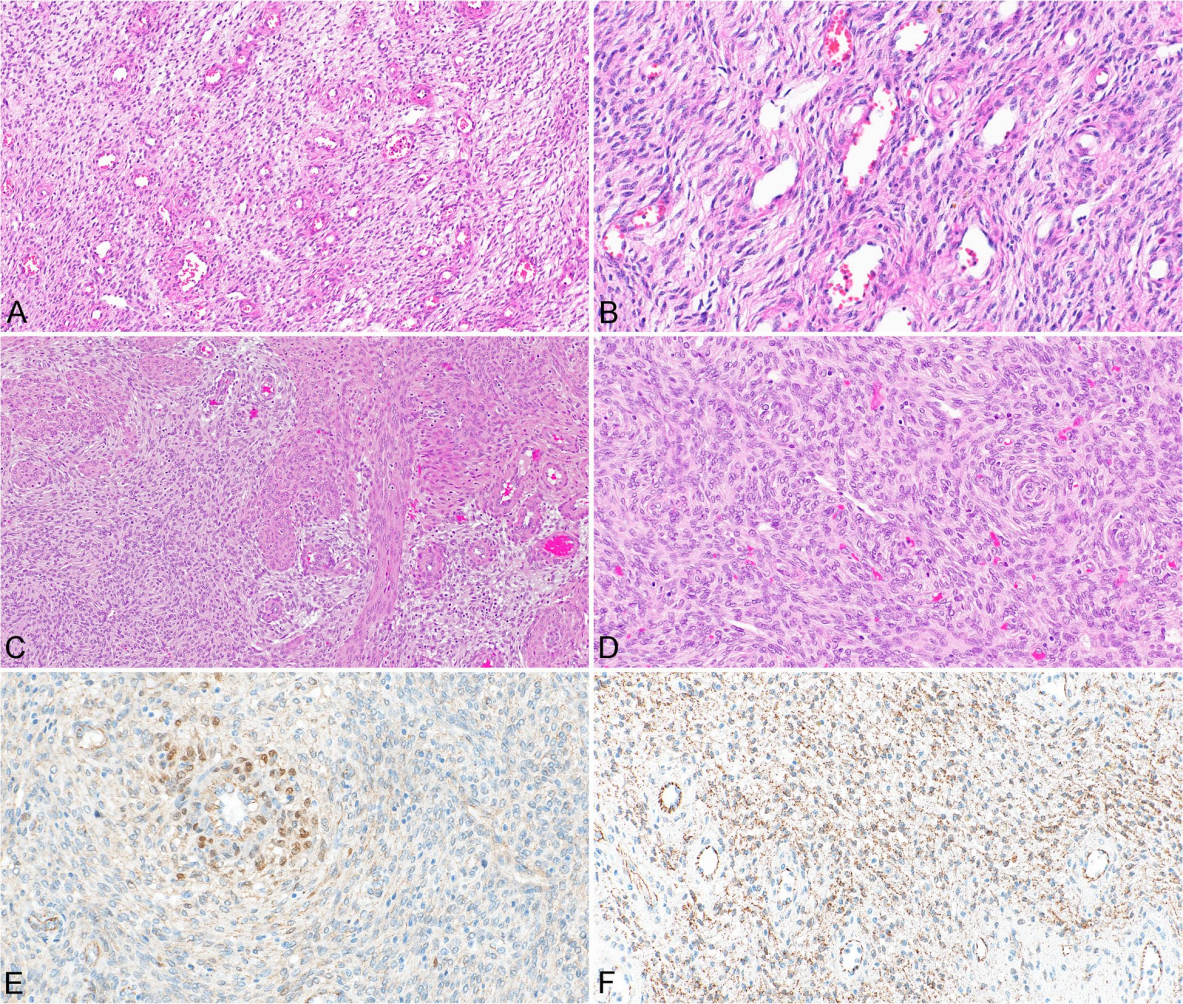
Fibroblastic variant of low grade endometrial sarcoma with typical spiral arterioles (**A**) (case #1; × 100). Tumor cells showing regular nuclei and fibrillary cytoplasm (**B**) (case #1; × 200). LGESS showing infiltration into the surrounding myometrium (**C**) (case #2; × 100). Tumor cells with regular oval nuclei with some perivascular whorling (**D**) (case #2; × 200). Immunohistochemical positivity of β-catenin with nuclear accumulation in tumor cells surrounding the spiral arteriole (**E**) (case #2; × 200). Granular cytoplasmic positivity of β-catenin (with nuclear negativity) (**F**) (case #1; × 200)

The resection specimen consisted of the uterine corpus divided into two parts containing a grey-white tumor measuring 30 mm in its greatest dimension. Microscopically, the tumor showed typical growth features of LGESS, with a “tongue-like” infiltration into the surrounding myometrium. The tumor consisted of spindle cells with mostly spindle-shaped and occasionally oval nuclei, showing only mild nuclear atypia (Fig. 1A, B). No mitotic figures were found. The cytoplasm was eosinophilic and focally fibrillar. Focal myxoid stromal changes were present. Small arterioles typical of LGESS were also identified. There was no lymphovascular space invasion and no involvement of the serosa. The tumor was in keeping with a fibroblastic variant of LGESS. The cervix and adnexa were grossly and histologically normal.

Immunohistochemically, the tumor was diffusely positive for CD10, IFITM1, estrogen receptor, and progesterone receptor. Variable expression of WT1, smooth muscle actin, and desmin was noted. Other smooth muscle markers, such as caldesmon, calponin, and transgelin, were negative. β-Catenin (both clones) showed weak cytoplasmic granular positivity without nuclear expression (Fig. 1F).

#### Case 2

The resected material from the laparoscopic myomectomy consisted of multiple tissue fragments (weight 41 g) which were grey-white in color. Microscopically, a typical LGESS was identified, showing irregular infiltration into the surrounding myometrium but no lymphovascular space invasion. The tumor consisted of oval and spindle cells mostly with round-to-oval nuclei and small nucleoli (Fig. 1C, D). There were minor areas with perivascular whorling (Fig. 1D). Tumor cells were monomorphic without significant atypia. The mitotic count was 3 mitoses per 10 high power fields (HPF). Typical small arterioles were present. The subsequent resection specimen showed no residual tumor, and the cervix and fallopian tubes were grossly and histologically normal.

Immunohistochemically, the tumor cells showed strong, diffuse expression of WT1 and CD10, IFITM1, estrogen receptor, and progesterone receptor. Focal and weak expression of desmin and calponin was present. Other smooth muscle markers examined, including transgelin and caldesmon, were negative. Focal nuclear expression of β-catenin was observed (both clones), mostly in the perivascular areas with whorling (Fig. 1E).

#### Case 3

**Fig. 2:**
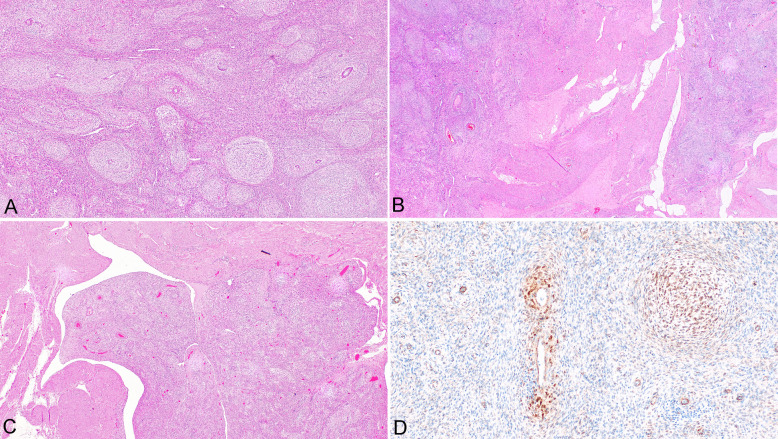
Endometrial stromal tumor with whorling (case #3). Whorls of tumor cells (**A**) (× 100). Tongue-like infiltrative growth into surrounding myometrium (**B**) (× 20). Tumor growing into large vessel (**C**) (× 20). Focal nuclear positivity of β-catenin (**D**) (× 200)

The resected material consisted of a uterus partly divided into two halves by the clinician. There was an irregular tumor mass in the dorsal and caudal part of the uterine corpus measuring 6 × 4.5 × 2.5 cm, infiltrating the myometrium. Microscopically, the tumor consisted of spindle or oval cells with regular nuclei. No mitotic figures were found. The architecture was characterized by a mixture of variably sized whorls, which were present in more than 90% of the tumor mass (Fig. 2A). Between these whorls, the tumor consisted of cells with the same features, but without whorling. In some of the whorls, small arterioles were present. Typical growth of LGESS was identified, with irregular infiltration into the surrounding myometrium (Fig. 2B). Focal lymphovascular space invasion was present (Fig. 2C). Immunohistochemically, the tumor cells showed strong, diffuse expression of estrogen and progesterone receptors. Variable expression of WT1, CD10, IFITM1, desmin, smooth muscle actin, and transgelin was present. Other smooth muscle markers, including calponin and caldesmon, were negative. Focal membranous and diffuse granular cytoplasmic expression of β-catenin (both clones) was present in tumor cells, but nuclear expression of β-catenin was observed only focally, mostly in whorls (Fig. 2D).

### Molecular findings

All three tumors lacked recurrent fusion events. All cases were microsatellite stable, and in all cases, a *CTNNB1* mutation was detected; in two cases, this was the only molecular event identified. In the first case, a likely pathogenic oncogenic *CTNNB1* mutation NM_001904.4:c.94G>T, p.(Asp32Tyr) was detected, with a variant allele frequency of 40% and a read depth of 962×. Copy number variation (CNV) analysis revealed no amplifications or deletions. In the second case, a pathogenic in-frame *CTNNB1* deletion of seven amino acids, NM_001904.4:c.125_145del, p.(Thr42_Gly48del), was detected, with a variant allele frequency of 17% and a read depth of 197×. CNV analysis could not be reliably evaluated due to insufficient sample quality. In the third case, a likely pathogenic oncogenic *CTNNB1* missense mutation NM_001904.4:c.122C>T, p.(Thr41Ile), VAF 48%, and a read depth of 344× was detected. In this case, aside from the *CTNNB1* mutation, a likely pathogenic truncating mutation in *MST1* (NM_020998.3:c.701del, p.(Pro234fs), VAF 48%), coding for an intracellular kinase, was also detected, and CNV analysis revealed a heterozygous deletion of *BARD1*, and amplification of *COL1A1* (6 copies) and of a part of the *TBX3* gene (exon 1–6 out of 8 exons; 8 copies).

**Fig. 3:**
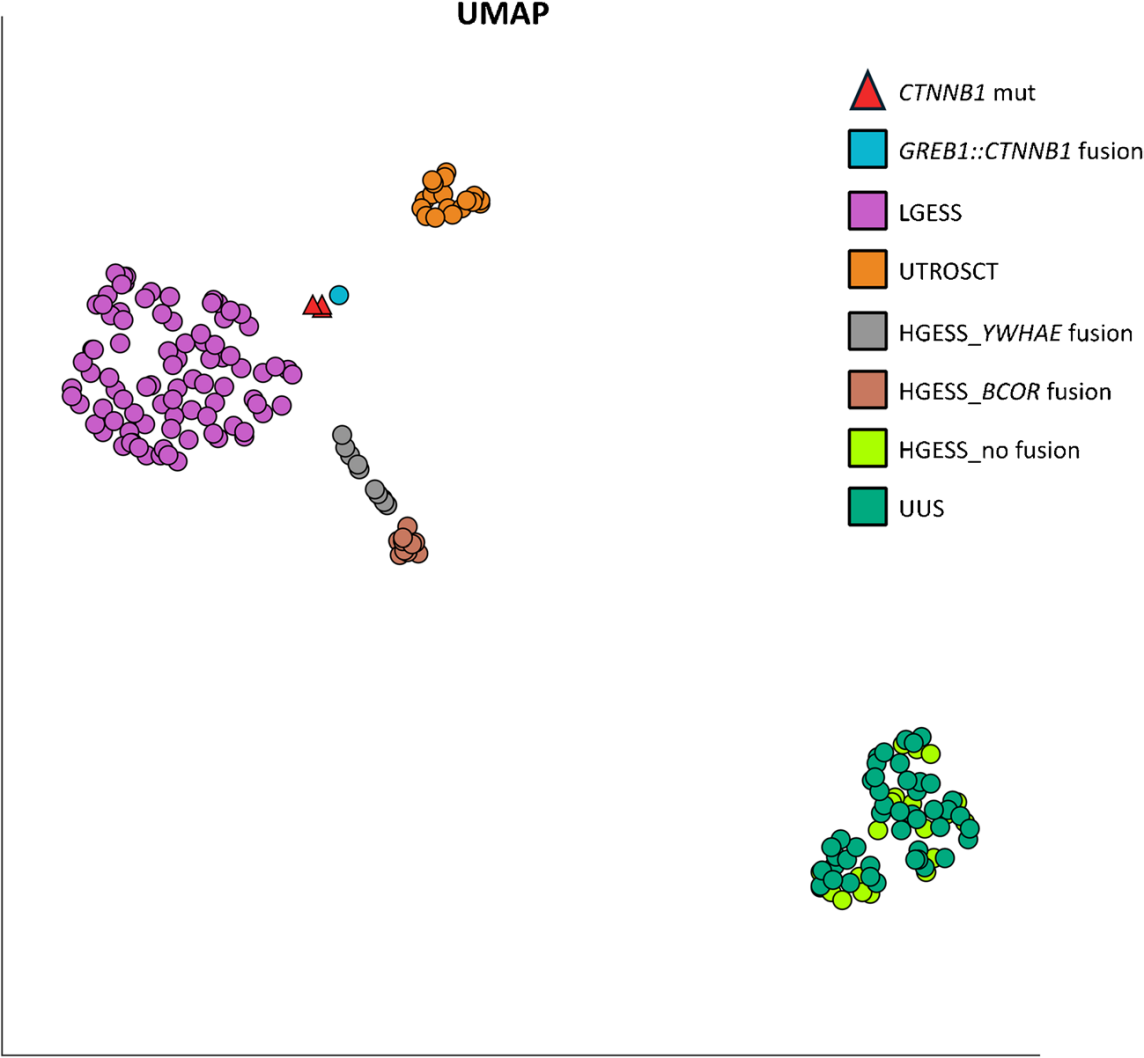
Transcriptomic profiling. Cluster analysis (1670 differentially expressed genes (*p* < 0.0001); UMAP) demonstrated a similar transcriptional pattern of three cases of endometrial stromal tumors (EST) with a *CTNNB1* mutation (red) and one case with a *GREB1::CTNNB1* fusion (blue), but distinct from the fusion-driven LGESS (87 cases; violet); fusion-driven UTROSCT (20 cases; gold) and highly aggressive HGESS—*BCOR*-fused (10 cases; brown); *YWHAE*-fused (10 cases; grey); HGESS cases without a drive fusion event (20; light green) and UUS (45; dark green)

RNA-Seq-based clustering analysis of the three *CTNNB1*-mutated cases and a previously described *CTNNB1*-fused tumor and a set of 192 uterine tumors revealed all four *CTNNB1*-altered cases clustering together between LGESS and UTROSCT cases closer to the LGESS cluster (Fig. 3).

**Fig. 4:**
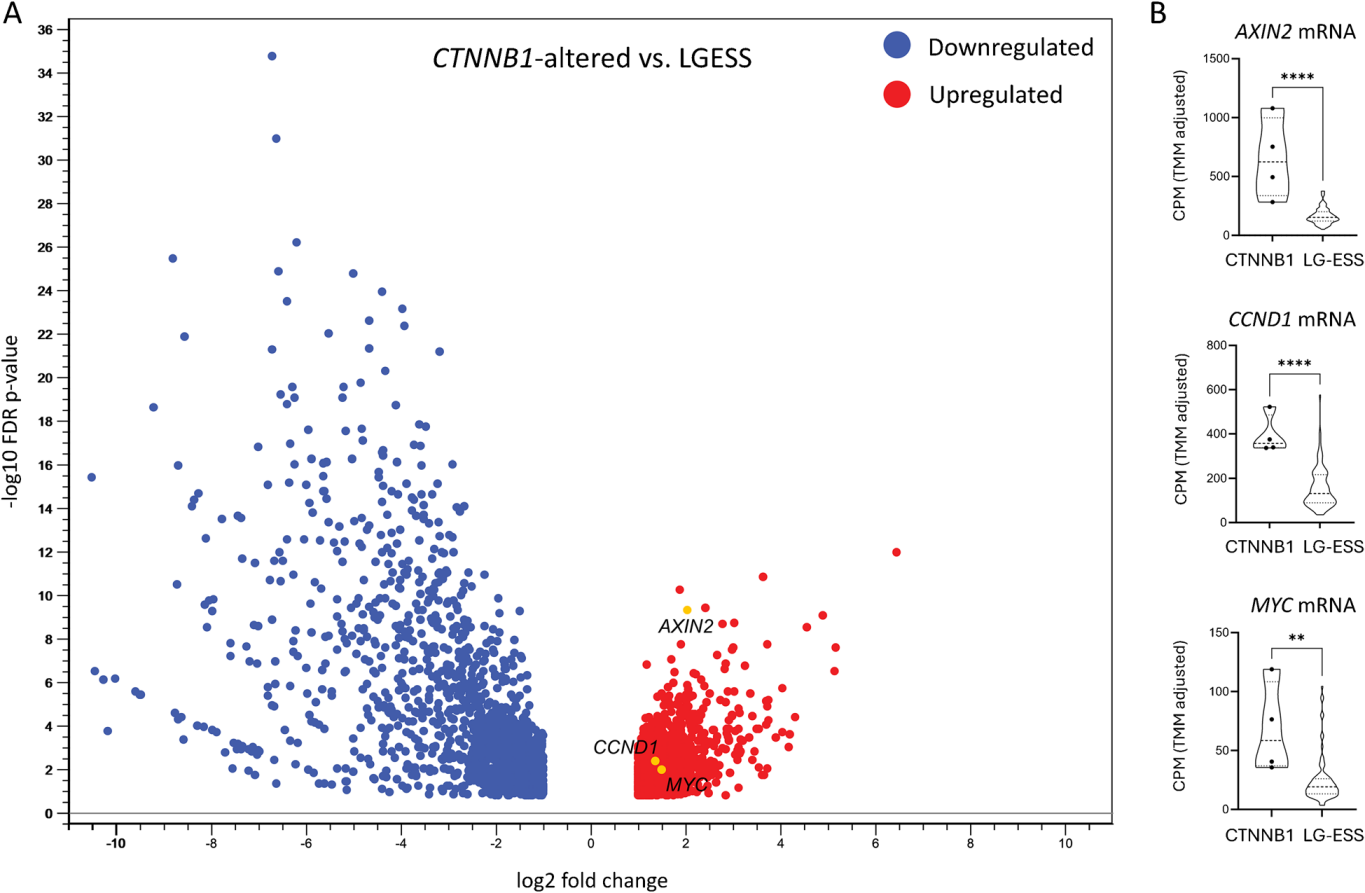
Differential gene expression analysis. RNA-Seq data were used to compare expression profiles of four *CTNNB1*-altered cases versus 87 LGESS. **A** A volcano plot depicting -log10 False Discovery Rate (FDR) versus log2 fold change difference in RNA abundance in transcripts per million reads (TPM). The TPM mean in max group > 5 and fold change > 2 were included in the analysis. **B** Comparison of TMM-normalized expression (counts per million; CPM) of *AXIN2*, *CCND1*, and *MYC*

Differential expression analysis of the 4 *CTNNB1*-altered tumors compared to the 87 LGESS showed 1486 significantly upregulated and 1562 downregulated genes based on the set parameters (Supplementary Table 1). Moreover, significantly upregulated mRNA expression of *AXIN2* (fold change = 4.07; FDR *p* < 0.0001), *CCND1* (fold change = 2.56; FDR *p* = 0.0043), and *MYC* (fold change = 2.81; FDR *p* = 0.0104) in the *CTNNB1*-altered group was observed (Fig. 4).

## Discussion

Wnt signaling pathway is involved in several processes, including modulation of differentiation during embryonic development, cell proliferation and division, epithelial-mesenchymal transition, and also cell-to-cell adhesion and extracellular signaling [16]. This pathway plays a crucial role in the pathogenesis of several tumors, including endometrioid carcinoma (ovary and endometrium), desmoid tumor, hepatocellular carcinoma, and colorectal carcinoma [17–20]. It can be altered by mutations in several genes, among which *APC* and *CTNNB1* play key roles. However, Wnt pathway dysregulation can also occur independently of *CTNNB1* (or *AKT*) mutations and may result from alterations in upstream genes, miRNA regulation, or epigenetic mechanisms [18, 21].

In most cases of LGESS, alteration of the Wnt pathway appears to be a driver event in tumor pathogenesis. Nevertheless, this alteration is usually not associated with *CTNNB1* mutation but rather with abnormal Wnt signaling caused by the fusions typical for the majority of LGESS, most commonly involving *JAZF1*,* PHF1*, and *EPC1* genes [7, 8]. Recurrent fusions occur in approximately 70–75% of LGESS; however, there remain cases in which the molecular alteration is unknown [2, 3, 15]. In these tumors, other molecular alterations, including gene mutations, may play a role, but available DNA sequencing data in LGESS are limited. Despite approximately 300 molecularly defined LGESS cases with recurrent fusions reported in the literature to date, data on their mutational profile remain scarce, as most studies have focused only on RNA-level alterations [3]. Only a few small studies have selectively analyzed *CTNNB1* mutations. Altogether, 31 cases in three studies have been evaluated, but no *CTNNB1* mutations were identified [8, 9, 22]. Only one case report described a LGESS with a *CTNNB1* mutation, and another study described one endometrial stromal nodule (ESN) harboring a *CTNNB1* mutation [9, 11].

Mutations of *CTNNB1* typically occur in exon 3, affecting phosphorylation sites critical for β-catenin ubiquitin-mediated degradation, thereby leading to its nuclear accumulation [18]. In our three cases and the two previously described cases (LGESS and ESN), the *CTNNB1* mutation was located in exon 3 [9, 11]. However, in the AACR project GENIE public dataset (GENIE Cohort v18.0-public; accessed 27 August 2025 through cBioportal, genie.cBioportal.org), *CTNNB1* mutations were identified in 2 of 53 uterine LGESS cases—one in exon 3 and the other in exon 7 [23]. Specifically, one metastatic LGESS harbored the p.(G34V) *CTNNB1* mutation in exon 3 (with amplification of chromosome 8p), while one local recurrence carried the p.(I357V) *CTNNB1* mutation in exon 7 (along with mutations in *TP53*, *ATM*, *BRD4*, and *CREBB*), which forms an Armadillo/beta-catenin-like repeat necessary for ligand binding. Neither case showed a recurrent gene fusion. Interestingly, in our three cases, there was no nuclear immunoreactivity with β-catenin in one case and only focal nuclear staining in the other two; this illustrates the important point that *CTNNB1* mutations are not always associated with diffuse nuclear β-catenin immunoreactivity.

In one of our cases, other alterations were also detected along with the *CTNNB1* mutation. Specifically, the *MST1* truncating mutation, and copy number changes resulting in deletion of *BARD1*, and amplification of *COL1A1* and of a part of *TBX3* were detected at the DNA level. The *MST1* gene is a signaling kinase of the Hippo signaling pathway, and its inactivation has been described as a secondary event in sarcomas [24].

It should be noted that not only *CTNNB1* mutations but also gene fusions involving *CTNNB1* can occur in some uterine mesenchymal tumors, including uterine tumor resembling ovarian sex cord tumor (UTROSCT) and the recently described entity endometrial stromal tumor with whorling and *GREB1::CTNNB1* fusion [12–14, 25]. There have been five reported cases of uterine tumors with whorling and *GREB1::CTNNB1* fusion [12–14]. Four of these tumors were well demarcated from the surrounding myometrium and, based on this, were regarded as probably benign. The fifth reported case was located in the parametrial tissue, without further description. One of our cases showed the same morphological features, with whorling present in more than 90% of the tumor mass. In another case, whorling was present focally in a perivascular location, but this pattern is not unusual for LGESS or ESN and the peculiar morphology with many variable sized whorls was not found. Contrary to the previously reported cases with *GREB1::CTNNB1* fusion, our case with whorling showed a typical growth pattern of LGESS with tongue-like invasion into the myometrium and lymphovascular space invasion. Based on our findings, we assume that alterations of the *CTNNB1* gene, either mutations or fusions, can result in a peculiar morphology characterized by a prominent whorling pattern, which is evidenced by the five previously reported cases as well as one of our cases. Based on this, we suggest that tumors with this morphology and alterations likely belong to the category of endometrial stromal tumors and their biological potential should be assessed according to the standard morphological criteria used for distinguishing between ESN and LGESS.

However, in the context of the results of transcriptomic profiling, which showed the three *CTNNB1*-mutated cases clustering very close to the *CTNNB1*-fused tumor but forming a separate cluster from the LGESS cases, we cannot exclude with certainty the possibility that tumors with *CTNNB1* alterations represent a separate entity in which the *CTNNB1* alteration is a driver event. Nevertheless, we also need to mention the limitations of this differentiation based on gene expression. Since the alteration in *CTNNB1*, a co-activator of transcription factors, affects the expression of other genes, it influences the expression landscape, and thus also the clustering analysis. Importantly, the upregulation of *MYC*, *CCND1*, and *AXIN2* supports a driver effect of the detected *CTNNB1* alterations in our cases.

Concerning the differential diagnosis of these tumors, one should be aware that a whorling architecture may be present in other uterine mesenchymal neoplasms rarely, including UTROSCT, and this pattern is not specific for ESN or LGESS with a *CTNNB1* alteration. Moreover, one case of UTROSCT with a *GREB1*::*CTNNB1* fusion has been described, but this case showed typical morphological features of UTROSCT [25]. This emphasizes the general rule that molecular findings must in each case be correlated with the morphological features and that different tumor types may exhibit the same molecular abnormalities. Finally, the expression clustering analysis does not include all uterine tumor types; therefore, we cannot exclude the possibility that other entities may show an even more similar transcriptomic profile.

In conclusion, our study shows that *CTNNB1* mutation can be a rare isolated molecular event in ESTs and probably represents a driver event in these tumors; this is supported by the absence of other molecular aberrations in most cases and observed mRNA upregulation of several key beta-catenin targets. The exact frequency of *CTNNB1* mutation in these tumors remains unknown, as most studies on LGESS focus on RNA sequencing. From a practical perspective, this finding suggests that a sole *CTNNB1* mutation, without any additional molecular changes, may be a feature of some LGESS/ESN and could be useful in differential diagnosis; however, more data are needed to confirm this observation. In addition, the results of our study show that a *CTNNB1* mutation can result in a peculiar morphological pattern with whorling. We believe that endometrial stromal tumors with *GREB1::CTNNB1* fusion and whorling, and tumors with a *CTNNB1* mutation represent part of the histological spectrum of ESTs (ESN or LGESS) and can be regarded as a separate category of these neoplasms characterized by a *CTNNB1* alteration as a driver event. However, more data with the reporting of additional cases are needed to elucidate this issue. Moreover, it is worth mentioning that Wnt signaling is the most commonly altered pathway in LGESS, suggesting that therapeutic targeting of this pathway could be beneficial in selected patients. However, despite ongoing research, there is currently no approved treatment specifically targeting the Wnt pathway or abnormal β-catenin expression [26, 27].

## Supplementary Information

Below is the link to the electronic supplementary material.

MOESM1 (XLXS 508 KB)

## Data Availability

The datasets used and/or analyzed during the current study are available from the corresponding author upon reasonable request.
